# Technology Use for Home-Based Stroke Rehabilitation in Switzerland From the Perspectives of Persons Living With Stroke, Informal Caregivers, and Therapists: Qualitative Interview and Focus Group Study

**DOI:** 10.2196/59781

**Published:** 2024-07-18

**Authors:** Lena Sauerzopf, Andreas Luft, Valeria Maeusli, Verena Klamroth-Marganska, Michael Sy, Martina Rebekka Spiess

**Affiliations:** 1 Institute of Occupational Therapy School of Health Sciences ZHAW Zurich University of Applied Sciences Winterthur Switzerland; 2 Faculty of Medicine University of Zurich Zurich Switzerland; 3 Division of Vascular Neurology and Neurorehabilitation Department of Neurology University of Zurich Zurich Switzerland; 4 Rehaklinik Bellikon Bellikon Switzerland

**Keywords:** home-based therapy, technology-based tools, app, stroke, outpatient rehabilitation, occupational therapy, physiotherapy, mobile phone

## Abstract

**Background:**

Stroke is a leading cause for long-term disability, requiring both inpatient and outpatient rehabilitation and self-training in the home environment. Technology-based tools are gradually gaining acceptance as additional and suitable options for extending the rehabilitation process. While the experiences of persons living with stroke, therapists, and informal caregivers with respect to technology use have already been investigated in other countries, this topic is underexplored in the Swiss context.

**Objective:**

We aimed to explore the experiences and needs of persons living with stroke, informal caregivers, and therapists in using technology-based tools in a home environment for stroke rehabilitation in Switzerland.

**Methods:**

This study followed a qualitative descriptive methodology, including semistructured interviews and focus group discussions. We applied a deductive template analysis alongside the accessibility, adaptability, accountability, and engagement framework to analyze the qualitative data sets for technology-assisted solutions for poststroke rehabilitation.

**Results:**

We collected the experiences and needs of persons living with stroke (7/23, 30%), informal caregivers (4/23, 17%), and therapists (occupational and physical therapists; 12/23, 52%). The 4 categories we used to organize the analysis and results were *accessibility to quality rehabilitation*, *adaptability to patient differences*, *accountability or compliance with rehabilitation*, and *engagement with rehabilitation*. Persons living with stroke stated that they use various tools within their rehabilitation process depending on their specific needs. They felt that there is a plethora of tools available but sometimes felt overwhelmed with the selection process. Informal caregivers indicated that they generally felt underserved and insufficiently informed throughout the rehabilitation process. They reported that they use technology-based tools to support their relatives affected by stroke in becoming more independent. Therapists appreciate the numerous possible applications of technology-based tools in rehabilitation. At the same time, however, they express dissatisfaction with the lack of clarity in Switzerland regarding cost coverage, recommendations, and training opportunities.

**Conclusions:**

Persons living with stroke, informal caregivers, and therapists in Switzerland reported varied and unique experiences and needs with the use of technology-based tools in outpatient stroke rehabilitation. Written recommendations, the assumption of financial costs, and the provision of information and education could foster increased confidence in the use of technology-based tools for patients and therapists.

## Introduction

### Background

Persons living with stroke frequently face persistent limitations in various domains, such as motor function and cognition, influencing daily life activities and participation [1]. Therefore, a considerable proportion of these individuals require long-term outpatient and home-based therapeutic interventions [2]. Previous research highlights that the use of technology-based tools at home can serve as a means to complement non–technology-based therapy in stroke rehabilitation [3].

For example, mobile apps are often used in combination with wearable sensors to increase therapy intensity and adherence to home exercise programs [4-6]. Other technologies, such as virtual reality serious games, augmented reality scenarios, and wearable sensors, allow the asynchronous monitoring of the recovery process and synchronous connections between health care providers and patients and provide education for clients or informal caregivers [7,8]. These examples illustrate the versatile use of technology-based tools for stroke rehabilitation for the home setting.

Previous research also indicates that such rehabilitation services, delivered through information and communication technology (ICT) and technology-based tools, result in comparable outcomes to those achieved through non–technology-based rehabilitation [9]. This leads to the fact that professional associations and health organizations advocate for the use of technology-based tools in rehabilitation [10-12]. Specifically, in their Regional Digital Health Action Plan for the European Region 2023-2030, the World Health Organization supports the continuous promotion and expansion of digital solutions to enhance health outcomes for all individuals and to push forward digital transformation [12,13].

### Context of Practice

The needs and experiences of at least occupational and physical therapists and survivors of stroke with regard to technology-based tools for the home-based setting have been investigated in other countries already [14-16].

One study [14] explored how physical and occupational therapists in Denmark view using ICT, such as apps, in stroke rehabilitation. They found that ICT could improve communication, documentation, and overall rehabilitation by empowering survivors of stroke and caregivers, facilitating follow-up care, and enhancing communication across sectors [14]. This study delved into the design needs for at-home poststroke rehabilitation robots in Ontario, Canada, contrasting perspectives between survivors of stroke and therapists. Through interviews with both groups, key design recommendations, potential features, and barriers emerged, highlighting the importance of incorporating the insights of survivors of stroke into home environments and therapists’ expertise in therapy methodology and safety. The findings underscored the necessity of tailored design approaches that consider a range of impairments, incorporate household items, and address individual motion requirements [15]. Another study [16] investigated Swedish health care professionals’ use of ICT for person-centered stroke rehabilitation. Findings suggest that integrating ICT could enhance collaboration between patients and therapists, as well as patient participation, guiding the development of a multidisciplinary intervention [16].

In Switzerland, the health care system is characterized by its federalist structure and combines both private and public elements. The quality of this health care system is considered very high [17]. However, the digitalization of health care, including rehabilitation, is still in its early stages [18]. Traditionally, inpatient stays in acute hospitals in Switzerland have been comparatively long, averaging 17 days [19]. Recently, a shift to a shorter length of inpatient stays and earlier outpatient treatment is visible as a trend to counteract high and increasingly high health care costs [18]. Because of this transition, we need new solutions. The use of technology-based tools is one possibility. This transition holds potential for the use of technology-based tools in outpatient and home-based stroke rehabilitation.

For the successful development and implementation of technology-based instruments, the needs and experiences of relevant groups are essential and should be considered. Research indicates that technology adoption depends on the perceived utility of the target groups [20]. However, today, most health care technologies are still designed *for* the target group rather than *cocreated with* the target group, leading to reduced rates of technology uptake [21]. A true user-centered, cocreative design approach emphasizes the relevance of investigating the experiences and needs of the person who will use these tools. In health care, and thus in stroke rehabilitation, we have the special case of having several user groups. Users include not only persons living with stroke and therapists (eg, occupational and physical therapists) but also informal caregivers. Informal caregivers are, for example, spouses, partners, and (adult) children. These are individuals who often provide their support without or with minimal reimbursement and specialized education. Their responsibilities span a wide range of tasks, from offering basic aid in daily activities (instrumental caregiving) to playing more complex roles such as coordinating health care requirements [22].

The lack of research on the use of technology in home-based stroke rehabilitation in Switzerland poses a challenge to obtaining a comprehensive understanding of the specific needs, experiences, and potential benefits and barriers in the Swiss context. It is unclear how far the needs and experiences of involved individuals in Switzerland are similar to those in other countries (translate over different health systems).

In this study, we aim to investigate the needs and experiences of persons living with stroke, therapists, and informal caregivers with regard to the use of technology-based tools in home-based stroke rehabilitation in Switzerland. We aim to provide a basis for the user-centered development of technology-based tools to support home-based stroke rehabilitation. Therefore, in this study, we sought to answer the following research question: What are the experiences and needs of persons living with stroke, informal caregivers, and therapists in using technology-based tools in home-based stroke rehabilitation within the Swiss context?

## Methods

### Design

We chose a qualitative, descriptive methodology approach using semistructured interviews and focus group discussions. This study followed a deductive template analysis (TA), in which the qualitative data sets were analyzed using the so-called *accessibility*, *adaptability*, *accountability*, and *engagement* (A3E) framework for technology-assisted solutions for poststroke rehabilitation [23-25]. We considered the A3E framework to be appropriate for the aim of our study, as the themes are addressing existing barriers in delivering technology-assisted stroke rehabilitation and potential solutions to enhance stroke rehabilitation through technology [17]. While inductive content analysis is used when no previous research has dealt with the phenomenon, deductive content analysis is used, for example, when an existing theory is tested in a new situation [26]. Because we are referring to an existing framework and comparable research has already been conducted in other geographical contexts, we considered a deductive approach to be suitable for this study. Furthermore, the pragmatic design of this approach makes it suitable for questions regarding the health environment and descriptions of the experiences and needs of the target group [27,28].

### Participant Selection

The sample comprised 3 cohorts: persons living with stroke, informal caregivers of persons living with stroke, and therapists of persons living with stroke. All participants from these 3 cohorts were required to speak Standard German or Swiss German and provide informed consent to participate in the study. We included persons living with stroke if they (1) were aged >18 years, (2) had a history of stroke in the past, (3) were currently living in a home-based setting, (4) were currently undergoing or had undergone outpatient therapy, and (5) were able to participate in an interview or discussion lasting at least 30 minutes. We included informal caregivers of persons living with stroke if they (1) were adult informal caregivers, (2) were currently living in the same household with a person living with stroke, and (3) were currently or had been involved in the outpatient rehabilitation process of this person living with stroke. Therapists were needed to (1) have experience in the treatment of persons living with stroke, (2) work in outpatient rehabilitation, and (3) have a professional background as occupational therapists or physical therapists or in a related therapeutic field (eg, sports therapy).

We recruited participants using a combination of email and telephone outreach through snowball sampling, contacting the patient’s and informal caregiver’s organization, rehabilitation clinics, outpatient therapy practices, therapists, or personal contacts of persons of 1 of the 3 cohorts. We spread a call for participation through the newsletter of the ZHAW Zurich University of Applied Sciences, School of Health Sciences. Individuals interested in participating in the study were encouraged to contact the research team. Comprehensive details regarding the research project, data security, data storage, and data processing were provided.

We inquired about the participants’ preferences for data collection methods, providing options for both face-to-face and web-based settings. Most people in all cohorts expressed a preference for the web-based setting (20/23, 87%). In the end, only focus group 1 (3/23, 13%) of persons living with stroke took place in person at a rehabilitation clinic in Switzerland. In addition, we proposed the option of individual interviews. This possibility was frequently favored due to its flexibility. It was more compatible with the participants’ daily routines and other responsibilities, particularly those of informal caregivers. We arranged the focus groups based on their respective cohorts, intentionally avoiding mixing different groups. Our focus was to create an environment where all participants felt free to talk openly. Due to potential interdependencies among participants from the different cohorts (eg, between persons living with stroke and informal caregivers, between persons living with stroke and therapists, and between informal caregivers and therapists), this separation was considered necessary and appreciated by our participants.

### Data Collection

Focus group discussions and semistructured interviews were conducted between March 2023 and February 2024 by 1 moderator (LS) and 1 observer (MS), both experienced in conducting these procedures. The moderator guided the conversations using a semistructured discussion guide with open-ended questions. The questions in the discussion guide were adapted slightly depending on the cohort. An example of a semistructured discussion guide used for the cohort of persons living with stroke is shown in Textbox 1. Meanwhile, the observer took discussion notes, posted clarifying questions, and monitored compliance with the specified meeting agenda.

The discussions that took place on the web were conducted and recorded using web-based conference and meetings programs (Webex [Cisco] and Microsoft Teams). Before the discussions, we provided participants with written instructions on using the videoconferencing program. In the group of persons living with stroke, some participants were dependent on private support to set up the videoconference but managed to organize it. The discussions were conducted in a mix of Swiss German and Standard German, depending on the participant’s mother tongue. All participants, as well as the research team, were able to understand both Swiss and Standard German and responded in the language they preferred. We video and audio recorded the conversations and ensured confidentiality.

Focus groups discussions and semistructured interviews lasted between 46 and 90 minutes.

**Semistructured discussion guide.**

**Questions**

1. What technologies do you use in everyday life?

      1.1 For what daily activities do you use technology?

      1.2 What do you like about it?

      1.3 What don't you like about it?

      1.4 What have you found challenging?

      1.5 What have you experienced helpful?

      1.6 What technologies do you use in relation to health topics?

2. What does using technology in your daily life mean to you? How important is this use for you?

3. Which technologies have you already used in rehabilitation?

      3. 1 What do you like about it?

      3. 2 What don't you like about it?

      3.3 What have you found challenging?

      3.4 What have you experienced helpful?

4. If you could “imagine” an ideal product for home training, what would it look like?

### Data Analysis

**Figure 1:**
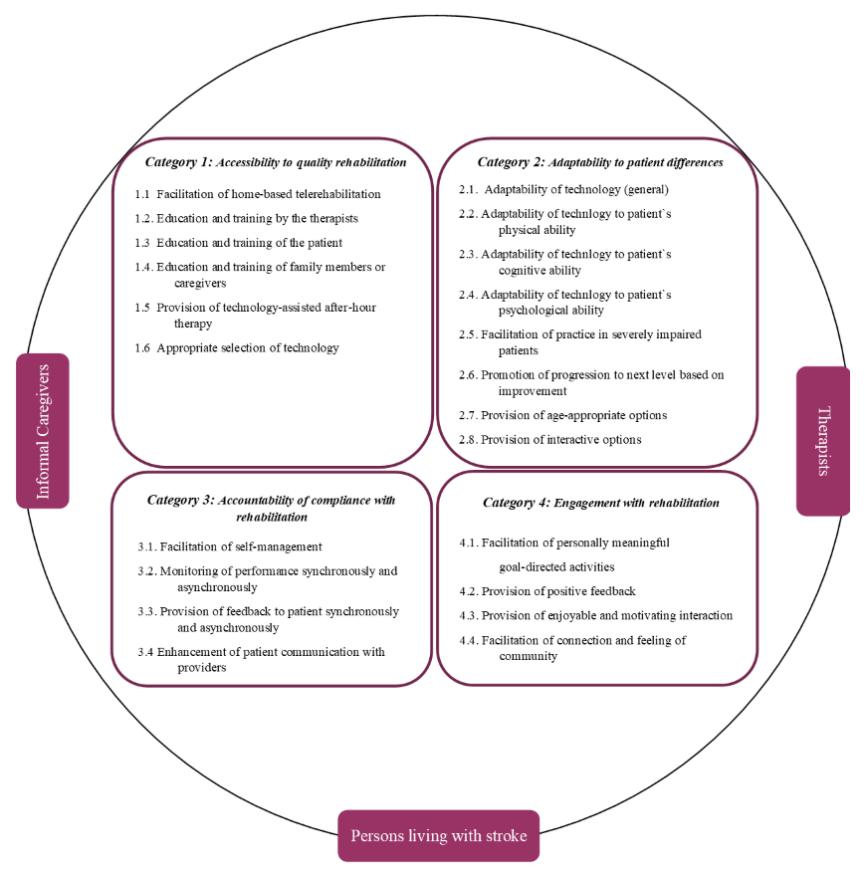
Adapted version of the accessibility, adaptability, accountability, and engagement (A3E) framework.

We transcribed the focus group and individual interviews in Standard German. The interviews conducted in Swiss German were also transcribed into Standard German. The translation of the quotes from Standard German to English took place during the preparation of this paper. We aligned the transcription following the simple rules put forth by Dresing and Pehl [29] and analyzed the data using the TA model for thematic analysis according to Brooks et al [23] with the software MAXQDA Analytics Pro (VERBI GmbH) [30]. We selected the technique of TA because it is generally highly flexible, although it follows a systematic approach, and allows researchers to customize the procedures to align with their specific requirements. Furthermore, it is an effective approach for investigating diverse perspectives of different cohorts within one common context. TA involves the creation and subsequent refinement of a coding template to represent the themes identified in the transcripts [25]. Our initial template was based on the A3E framework for technology-assisted solutions for poststroke rehabilitation, consisting of the following four major themes: (1) accessibility to quality rehabilitation, (2) adaptability to patient differences, (3) accountability or compliance with rehabilitation, and (4) engagement with rehabilitation [24]. We adopted the 4 major themes of this framework as categories. Furthermore, we adapted the subcategories based on the original model developed by Jayasree-Krishnan et al [24] and used them as our themes. We adjusted the wording of the subcategories and themes accordingly. Furthermore, we split some subcategories or themes that were rich and diverse in content into multiple subcategories or themes. An illustration of the adapted version of the A3E framework is displayed in Figure 1.

We proceeded in the following adapted steps [25]. The first step was the definition of “a priori” themes based on the A3E framework [24]. Afterward, we conducted an initial coding of a subset of the first 3 transcripts by assigning text sections to the a priori themes, including the rejection and modification of preliminary themes. The third step included the application of the template to the full data set.

### Rigor and Trustworthiness

To maintain rigor and trustworthiness, we adhered to the principles outlined by Nowell et al [31]. To ensure credibility, the participants had the option to pose questions and clarify their responses. In addition, we verified the accuracy of our transcriptions by comparing them to the original recordings. Furthermore, we incorporated peer debriefing as external feedback, seeking input from colleagues to ensure the rigor of our research process. We ensured transferability by applying a template derived from the A3E framework [24] that can be transferred to other socioeconomic systems and across various geographical regions. To strengthen dependability, we documented our research process, including the recruitment strategy and data analysis, in a clear and logical manner and relied on our documentation throughout the process.

### Ethical Considerations

We integrated ethical considerations into our research. Recognizing the far-reaching obligations of our participants and aiming to respect their limited time, we provided the option of participating in individual interviews alongside the focus groups to work around time constraints. We also submitted our research project to the cantonal ethics committee of Zurich (BASEC-Nr. Req-2023-00106). This committee determined that the research project does not fall within the scope of the Human Research Act and, therefore, does not fall within its remit. We explicitly informed participants about confidentiality concerns and their right to withdraw from the study at any point without stating reasons. Each participant gave a written declaration of consent. Study data, including identifying information and transcriptions, were deidentified.

### Positionality Statement

The first author, LS, is a doctoral student (Care and Rehabilitation Science), bringing a background and clinical experience as an occupational therapist. AL is a medical doctor, an academic, a researcher, and a rehabilitation clinic manager. VM is a master’s student (physical therapy, focusing on professional development). VK-M is a former academic and researcher currently working as a medical doctor in a rehabilitation setting. MS is an academic and a researcher and has a professional background in occupational therapy (OT) and occupational science. MRS is a professor, a rehabilitation scientist, and an academic researcher and a physical therapist by training. All authors have experience in researching technology in the context of stroke neurorehabilitation and are experienced in the Swiss health care system.

We as a team of authors cultivated a contextual constructivist position, recognizing the existence of multiple interpretations for any given phenomenon and depending on the contextual aspects of the research [32].

## Results

### Description of Participants

In total, we included 23 participants in this study. Of them, 12 (52%) participants were therapists (female: 12/12, 100%), 7 (30%) participants were persons living with stroke (female: n=3, 43%; male: n=4, 57%), and 4 (17%) participants were informal caregivers (female: n=3, 75%; male: n=1, 25%). Further details are provided in Table 1.

**Table 1:** Participant’s characteristics.

Cohort, focus group or interview, and ID				Sex (male, female, or intersex)		Country of residence		Canton or federal state of residence		Professional background
**Therapists**										
	**Focus group 1**									
		T1	Female		Switzerland		Zurich		OT^a^	
		T2	Female		Switzerland		Berne		OT	
		T3	Female		Switzerland		Berne		OT	
		T4	Female		Switzerland		Zurich		OT	
		T5	Female		Austria		Vienna		OT	
		T6	Female		Switzerland		Zurich		OT	
		T7	Female		Switzerland		Zurich		PT^b^	
		T8	Female		Switzerland		Berne		OT	
	**Focus group 2**									
		T9	Female		Switzerland		Zurich		OT	
		T10	Female		Switzerland		Zurich		PT	
		T11	Female		Switzerland		Zurich		ST^c^	
		T12	Female		Switzerland		Zurich		PT	
**Persons living with stroke**										
	**Focus group 1**									
		S1	Male		Switzerland		Thurgau		—^d^	
		S2	Female		Switzerland		St Gallen		—	
		S3	Male		Switzerland		Thurgau		—	
	**Interview 1**									
		S4	Male		Switzerland		Fribourg		—	
	**Interview 2**									
		S5	Male		Germany		Bavaria		—	
	**Interview 3**									
		S6	Female		Switzerland		Thurgau		—	
	**Interview 4**									
		S7	Female		Switzerland		St Gallen		—	
**Informal caregivers**										
	**Focus group 1**									
		C1	Female		Germany		Baden-Wurttemberg		—	
		C2	Female		Switzerland		Thurgau		—	
	**Interview 1**									
		C3	Female		Switzerland		Thurgau		—	
	**Interview 2**									
		C4	Male		Switzerland		St Gallen		—	

^a^OT: occupational therapy.

^b^PT: physiotherapy.

^c^ST: sports therapy.

^d^Not applicable.

The participants were residents of 5 different cantons of Switzerland and spoke Swiss German or Standard German: 35% (8/23) from Zurich; 22% (5/23) from Thurgau; 13% (3/23) from Berne; 13% (3/23) from St Gallen; and 4% (1/23) from Fribourg. Of the 23 participants, 2 (9%) were residents of Germany, and 1 (4%) lived in Austria. Participants who resided abroad had a strong connection to Switzerland and its health care system, such as undergoing rehabilitation treatment at a clinic in Switzerland, working within the Swiss health care system, or caring for a relative in Switzerland. All therapists who participated in the focus groups had experience in providing therapeutic support to persons living with stroke who were already living in the community.

Of the 7 persons living with stroke, 6 (86%) were of employable age (range 30-63 years). At the time of their stroke, these 6 persons living with stroke were actively employed, and 5 (83%) of them were also able to return to work after stroke. The sixth person living with stroke chose early retirement. The seventh person living with stroke, despite already being aged 79 years, self-identified as being professionally still active.

All informal caregivers included in the study described themselves as being employed.

### Thematic Analysis According to the A3E Framework

#### Overview

A pattern of cohort-specific needs and experiences with technology-based tools in outpatient stroke rehabilitation emerged. We classified these experiences into the 4 categories: *accessibility to quality rehabilitation*, *adaptability to patient differences*, *accountability of compliance with rehabilitation*, and *engagement with rehabilitation* [24]. Statements from participants are incorporated in this *Results* section. A detailed overview of additional quotes can be found in Multimedia Appendix 1. In the subsequent descriptions of the findings across the 4 categories, we have included the categorization into the corresponding subcategories or quotes within parentheses to enhance clarity.

#### Category 1: Accessibility to Quality Rehabilitation

Given that technologies are an option to provide appropriate access to rehabilitation, several topics regarding the accessibility to quality rehabilitation were discussed. Some therapists in our study reported that they had their first experience with synchronous telerehabilitation during the COVID-19 pandemic (subcategory 1.1 in Figure 1). Before that, the topic was not perceived as being present in the Swiss health care system. One of the therapists had already used telerehabilitation abroad. During the pandemic, several occupational and physical therapists found this option useful for conducting remote therapy sessions. However, not all participating occupational and physical therapists made use of this option. In addition to the finding that the provision of telerehabilitation services in the workplace was not feasible for some of the therapists, the tariff structure proved to be another significant challenge. Although the costs for providing occupational and physical therapy at a distance were covered during the COVID-19 pandemic, this was not consistently maintained for occupational and physical therapists afterward. None of the interviewed persons living with stroke or caregivers had any experience with synchronous telerehabilitation (subcategory 1.1 in Figure 1). However, there was interest in telerehabilitation; S7 expressed, “I think that [telerehabilitation] would surely make sense, but I don’t know if there’s an offer where I could do that” (original: “Also i dänk das [Telerehabilitation] wär sicher sinnvoll aber i wüsst gar nöd wos so eis Angebot gäbte”). The topics of education and training of the individual target groups regarding asynchronous technology-based tools for outpatient rehabilitation were discussed intensively in all interviews and focus groups, irrespective of the cohort. Overall, persons living with stroke preferred therapists (subcategory 1.2 in Figure 1) to take on the task of providing them with the relevant information. One of the participants said, for example, that it would be beneficial for him if the therapist could inform him about the available technology-based tools and their practical relevance in individual cases. Informal caregivers concurred with this perspective (subcategories 1.2 and 1.3 in Figure 1). They also perceived therapists as experts who should lead patients and assist them in the implementation of technology-based tools. One of the caregivers stated the following:

I’m the kind of a person who thinks that there are experts for that. I think it’s important for us caregivers, we already take on so much...Especially elderly caregivers. I already find our life so radically different; if we had to program the tool ourselves, it would be annoying...And I have realized, I can only speak for myself; it is much more effective if other people tell him something...That’s why I believe it’s more important for him to do it with therapists. (Original: I bin det dure eher so igstellt, dass i find für da gits Fachlüüt. I find wie für üs Aghörige wichtig, mer übernehmet bereits so viel...Au bei älteri Aghörige. I find üser Lebe is scho so krass andersch; wenn mer ez no muesset das Tool vilicht programmiere halbe, dann nervts einem vilicht au...I ha fescht gmerkt, i cha da nur für mi rede, es nützt viel mehr wenns andre Mensche ihm seget...Drum glaubi is es wichtiger, macht er da mit Therapeute.)

C3

All informal caregivers reported feeling overwhelmed by the transition, having to take on new tasks that were previously carried out by the persons living with stroke or jointly with them. Examples such as household budget planning, paying bills, and planning and making purchases were mentioned. Most therapists aimed to minimize the involvement of informal caregivers, recognizing the stress and pressure informal caregivers already endure (subcategory 1.4 in Figure 1). A topic that was prominently discussed in the focus groups and all interviews of the caregivers was the wish for more information on technology-based support devices and education on stroke rehabilitation in general to help them navigate the changes in their daily life with persons living with stroke. They expressed a strong need for education and training in this regard:

My husband was in rehab, and he had a full schedule. He sent me that, then I saw what he was doing. People were taking care of him. And I was at home, had four children, one is going through a tough puberty. Then nothing happened. So, I didn’t receive any information about the meaning [of his health condition] and how he would behave when he returns. (Original: Mi Maa isch in der Klinik gsi, er het immer voll Programm gha. Er het mers gschickt, damit i gseh was er macht. Ma het uf ihn glueget. I bin dihei gsi, ha det no vier Chind dihei gha, einer starch pubertierend. Denn isch eigentlich nüd cho. I han kei Info übercho wa bedüdtet da [seine Gesundheitssituation] und wie isch er denn.)

C1

Therapists found education and training for themselves and for individuals living with stroke and informal caregivers to be challenging. T2 expressed a need for action recommendations for using technology-based tools in rehabilitation. T10 felt reluctant to use technology due to the time commitment required for familiarization and training. Tasks such as this often have to take place outside of working hours, and therapists cannot claim any reimbursement for their time (subcategory 1.2 in Figure 1).

As for the appropriate selection of technology (subcategory 1.6 in Figure 1), therapists in both focus groups argued that the tool should be user-friendly, such as having the possibility of adaptations for different needs. Participating occupational therapists highlighted the use of everyday life tools that are already integrated into patients’ lives, such as tablets or smartwatches, alongside tools developed for therapeutic purposes. In addition, most therapists considered it crucial for persons living with stroke to use these tools as independently as possible to prevent conflicts and dependencies between them and their informal caregivers. Some therapists used the exchange at specialist meetings and with interest groups to help the therapists select appropriate tools. T6 stated that they [T6 and T1] had a specialist meeting the year before with the interest group technologies in OT, where they received many new ideas. T10 also added that she thinks it is important that the Swiss professional OT and physiotherapy associations recognize technology as part of the profession and that it is anchored accordingly in the tariff structure.

Several persons living with stroke stated that they were overwhelmed with the choice of technology-based tools (subcategory 1.6 in Figure 1), particularly with the selection and installation of training apps. For example, S6 expressed that, especially, the process of finding a suitable app from the app store was perceived as time-consuming, as well as evaluating the app in advance regarding its quality, which led to wrong purchases. Another person living with stroke stated that she had difficulty understanding the apps and was, therefore, unable to learn how to use them. Most of the persons living with stroke stated that the process should be simple and straightforward.

#### Category 2: Adaptability to Patient Differences

Among the persons living with stroke and therapists, the possibility to adapt the technology, in the sense of personalization, was considered as a relevant criterion for the selection of the tool. Some therapists expressed that to correctly and confidently personalize the technology to their patient, such as by selecting suitable exercises, they needed an extended phase of familiarization with the tool. This was perceived as time consuming and a hindrance to implementation (subcategory 2.1 in Figure 1):

I also needed a lot of time at the beginning...until I became familiar with it...It took a lot of clicks...you still have to individualize everything a bit. Yes, I was a bit afraid to really put it into practice. (Original: Also bi dem Tool hani am Afang au extrem viel Ziit bruucht...bis i selbscht es biz Routine gha han...Es het extrem viel Klicks brucht...Ma het immer no alles muesse individualisiere. Ja, da hani mi biz gschüüt am Afang davor das au würkli denn ind Praxis ine zneh.)

T12

Persons living with stroke encountered challenges in accessing and using a familiar technology, such as a PC or tablet, for outpatient stroke rehabilitation. For example, S7 reported that she was no longer able to switch on the PC and log in without help because her hand and arm function was impaired (subcategory 2.2 in Figure 1). She always needed to ask her husband for support. She also reported that she struggled remembering passwords (subcategory 2.3 in Figure 1). In some cases, persons living with stroke used the possibility to adapt the technology to accommodate for these difficulties. For example, persons living with stroke were able to use voice input and biometrical authentication on their technologies (subcategory 2.2 in Figure 1). In other cases, however, they rejected using these options, as they felt that they had to adapt to the new situation and gradually regain their ability to use of these tools (subcategory 2.1 in Figure 1). S2 stated, “I had the feeling that it [the rehabilitation process] takes time and that you have to accept it [own limitations]” (original: “I han mer sGfühl, es het Ziit brucht und eifach au es anneh, ez im Moment hanis usgschöpft”).

Therapists see technologies as providing some advantages, specifically for persons living with stroke with severe impairments (subcategory 2.5 in Figure 1). For example, some of the persons living with stroke live with severe impairments, making the journey to a clinic challenging to organize and conduct. Therapy intensity can then be increased by conducting parts of the therapy in the home environment, using technology-based tools. In facilitating the practice for these people, according to the experience of some of the therapists, informal caregivers play a crucial role. They regularly assist in the use of the technology-based tools, for example, in selecting the right exercises (subcategory 2.5 in Figure 1).

Both persons living with stroke and therapists valued the option that tools automatically adjust their levels (subcategory 2.6 in Figure 1). This was seen as an opportunity to shape the therapy. Furthermore, persons living with stroke appreciated the shaping of the therapy to their needs, such as gradually increasing the difficulty level according to their individual abilities. However, one of the persons living with stroke reported feeling overwhelmed even with the lowest level of a cognitive training program. A therapist noticed that she was familiar with cognitive training tools that automatically adapt but was unaware of tools for other functional areas, such as for motor training, that are available in Switzerland (subcategory 2.5 in Figure 1).

The topic of age-appropriate option (subcategory 2.7 in Figure 1) was briefly discussed. Some therapists had observed that older persons tend to face more challenges with videoconferencing tools, leading to a shift to telephone contact. The option of videoconferencing was used more often with younger persons:

The elderly patients said that [videoconferencing] was too complicated. We often just called them and asked how things are going and whether they performed their exercises... (Original: Die älteren Patienten sagten, das [Videokonferenzen] ist wie zu kompliziert. Wir haben dann oft einfach angerufen und gefragt wie’s geht und ob die Übungen möglich sind...)

T3

I did a few videoconferencing sessions...but they were all with younger patients after stroke. (Original: Ich habe einige Videokonferenz-Sitzungen gemacht...aber es waren alles jüngere Schlaganfallpatient:innen.)

T1

#### Category 3: Accountability or Compliance With Rehabilitation

Overall, persons living with stroke recognized the utility of technology-based tools in assuming responsibility for their own outpatient rehabilitation process (subcategory 3.1 in Figure 1). This was mainly due to the feedback they received from technology-based tools and autonomy they had in selecting individual exercises. S4 described the following experience (subcategory 3.2 in Figure 1):

After half an hour, you will have an evaluation on the screen of what you did wrong, what was good and what progress you have made. That is certainly good and motivating (Original: Auf dem Bildschirm hat man nach einer halben Stunde die Auswertung, was hast du recht falsch gemacht, was war gut, was hast du für Fortschritte gemacht. Das wär sicher sehr wichtig und motivierend auch)

Likewise, some of them found reminders to be helpful for staying on the rehabilitation track and for structuring their days in addition to therapy. A minority experienced limited additional benefits in their therapy process, primarily attributed to the absence of direct interaction with a therapist (subcategory 3.4 in Figure 1). S6 expressed difficulties in interpreting the scores of technology-based tools (subcategory 3.2 in Figure 1): “It is therefore inexplicable to me how it [the feedback] is created. I can’t understand the ratings” (original: “Also es ist mir unerklärlich, wie sich das [Feedback] zusammensetzt. Die Bewertungen kann ich nicht nachvollziehen”).

Informal caregivers viewed the assumption of personal responsibility by the persons living with stroke positively. One of the informal caregivers mentioned implementing specific tools to support his wife’s independence at home during the rehabilitation process. They purchased a smartwatch to enable her to make phone calls independently, which was not possible with a mobile phone at that time.

Therapists argued that it was important for them to enable the self-management of persons living with stroke when using technology-based tools (subcategory 3.1 in Figure 1). T10 shared the following experience: “My situation is like T12. We try to incorporate this [technology] into self-management, even if it is associated with limitations [of technology use]. I try to find a level at which the patient can still take responsibility for themselves” (original: “Bei mir ist es wahrscheinlich ähnlich wie bei T12. Wir versuchen es im Selbstmanagement einzubauen, auch wenn es mit Einschränkungen [der Technologienutzung] verbunden ist. Ich versuche dann lieber ein Level zu finden, bei dem der Patient Selbstverantwortung übernehmen kann.”). For these therapists, this includes enabling persons living with stroke to use the devices independently, that is, without the support of informal caregivers if possible.

The provision of feedback (subcategory 3.3 in Figure 1) was also mentioned by most therapists as a criterion to which they pay attention. For them, it was important to personally provide feedback to the persons living with stroke on the use and progress of technology-based tools. They also emphasized the importance of the tools themselves providing direct feedback when the persons living with stroke use them at home.

#### Category 4: Engagement With Rehabilitation

To maintain engagement in home-based rehabilitation, establishing personalized and meaningful goals is beneficial (subcategory 4.1 in Figure 1). Some of the persons living with stroke found it supportive to identify achievable goals that can be targeted during technology-supported therapy. Establishing meaningful goals allowed them to evaluate their progress and ensure that they are staying on track with their rehabilitation journey. For example, S7 elaborated that her goals include devising compensatory strategies for everyday life tasks, such as zipping her pants with one arm. She explained that to reach this goal, she frequently consults instructional videos on YouTube (Google LLC) for guidance. Therapists, especially some of the participating occupational therapists, expressed that they find it challenging to integrate technologies into reaching goals. For occupational therapists, it is essential that the specific goals align with the client’s daily life challenges. They regarded technologies as having the drawback of being constructed environments that, in their perspective, cannot be seamlessly integrated into individual everyday life. Occupational therapists questioned whether technologies could be used to achieve meaningful goals:

For me, it’s always a bit of the specific goal and that is simply always everyday-life oriented and individual at best. That’s why I’m also critical of technologies in the broadest sense, whether they can really do justice to the complexity of everyday life...[Goal setting including technologies] usually rather limited, as they are always constructed settings or are modeled on an everyday life situation...I think that’s the main limitation, it’s always an imagined reality. (Original: Für mi isch’s halt au immer biz e so d’Frog nach der konkrete Zielsetzig und die isch halt im beschte Fall scho immer alltagsorientiert und sehr individuell. Drum bin ich det scho kritisch gegenüber Technologie im wiiteschte Sinn, ob die denn würkli so dere Komplexität vom Alltag überhaupt chönt grecht werde...[Zielsetzung, die Technologien inkludiert] isch scho eher limitiert, es sind jo immer konschtruierti Settings, oder wo denn möglichscht irgendeinere Alltagssituation nachempfunde sind...I glaub scho chli das isch d Hauptlimitation, es isch immer e usdänkti Realität.)

T9

To address these challenges, some occupational therapists developed strategies (subcategory 4.1 in Figure 1). They used everyday life apps, such as public transportation apps or reminder apps, incorporating them into treatment planning and establishing links to daily activities:

What I always try to use are apps, calendar apps on the mobile phone, so that you can really concentrate on the activity. Or also the SBB app [public transportation]. I’ve practiced for hours with patients on reminder functions and apps, where you can make notes or something. The things that I also use myself. (Original: Was ich auch immer wieder versuche zu nutzen sind Apps, Kalenderapps auf dem Handy, um die Betätigung möglichst ins Zentrum zu rücken. Oder auch die SBB App [öffentliche Verkehrsmittel]. Da habe ich schon stundenlang mit Patienten geübt, oder auch irgendwelche Erinnerungsfunktionen und Erinnerungsapps, wo man sich auch Notizen machen kann oder so. Die Dinge ich halt auch selber benutze.)

T4

Most persons living with stroke experienced that positive feedback (subcategory 4.2 in Figure 1), such as good scores in a game, can contribute to a positive user experience. Persons living with stroke also frequently mentioned that they appreciate the option to personalize the technology-based tool in an appealing way (eg, color and background selection; 4.3). Negative feedback, such as bad scores in a game, by contrast, is perceived as demotivating (eg, the feeling of being too slow). Therapists concurred with this experience reported by persons living with stroke. They confirmed the positive effect of the incorporation of a reward system, the graphical representation of successes, or the presence of a checklist where completed exercises could be marked on motivation and adherence to therapy (subcategory 4.2 in Figure 1).

Persons living with stroke had controversial perceptions about the effect of technology-based therapy on human connections and their experience of community (subcategory 4.4 in Figure 1). S5 experienced practicing with technology-based tools as isolating, as he was missing the dialogue with people and, in his case, with therapists. S1 reported that the use of technology-based tools in his outpatient rehabilitation process brings him closer to his children, who played the training games together with him.

Informal caregivers reflected on their role in the rehabilitation process. All informal caregivers experienced a lack of community and a sense of belonging (subcategory 4.4 in Figure 1). They particularly felt abandoned during the transition from inpatient to outpatient rehabilitation. They had the impression that they were not adequately prepared by health care professionals for the changes coming their way due to their loved one’s condition. C1 described her situation as follows:

[I would have liked] more information about what to expect [all the bad things that could happen]. If this is not the case, you can be relieved...But it would give me the feeling that don’t have to do everything on my own. (Original: Eifach mehr Informatione, was chämti uf ei zuecho. Wenns denn nid so is, cha mer jo froh sii. Es gebti es Gfühl vo mer muessti nid alles allei mache.)

The included informal caregivers expressed that the opportunity to inform themselves as informal caregivers and possibly meet as a group could have been very helpful for them (subcategory 4.4 in Figure 1).

However, several informal caregivers expressed reservations about traditional informal caregiver support groups, as they believe these groups may not be constructive. The informal caregivers in our study associated these meetings with a tendency to wallow in self-pity within the caregiver support group. Nevertheless, most informal caregivers desired a constructive exchange and appreciated the opportunity to gain new perspectives from others facing similar situations. Technology could also be supportive in this context, as they would prefer a web-based format due to their numerous commitments. Informal caregivers perceived that technology could provide them with significant benefits here:

But now we know that all have a lot of commitments, things, or travel, and it has become more complicated. That’s why I’ve realized that it’s not so bad to just talk online. You can get into the topic very quickly. (Original: Aber inzwischen wir, wir haben alle sehr viele Pflichten, Sachen, oder Reisen und es ist komplizierter geworden. Deshalb merke ich auch, online einfach so Gespräche zu führen, ist auch gar nicht so schlecht. Man ist auch ganz schnell im Thema drin.)

C2

## Discussion

### Principal Findings

The objective of this study was to describe the experiences of persons living with stroke, informal caregivers, and therapists regarding the use of technology-based technology in home-based rehabilitation within the Swiss context. For this purpose, we conducted a deductive TA of the gathered data, presenting the findings following the A3E framework [24]. This approach provided a suitable foundation for reflecting influences as well as potential barriers pertinent to the Swiss health care system and society.

The participants in our study exhibited a generally positive attitude and high level of interest in the use of technology-based tools in home-based stroke rehabilitation. One of the persons living with stroke expressed criticism regarding the use of technology in therapy and everyday living.

The cohorts of persons living with stroke and informal caregivers were well mixed in terms of sex. All therapists identified as female. Furthermore, 8 (67%) out of the 12 therapists were occupational therapists. A nationwide survey revealed that 90% of occupational therapists in Switzerland identify as female [33], which indicates an acceptable sex distribution in the cohort of therapists.

The findings of our study suggest that informal caregivers of persons living with stroke in Switzerland face similar burdens to those identified in previous studies [14-16]. Participating informal caregivers also reported that dealing with the behavioral and personality changes of their family members posed a challenge for them.

Taking on activities and tasks that were previously the responsibility of the family member affected by stroke, such as household budget planning or paying bills, and the associated change of role were perceived as stressful. All informal caregivers who participated in our study reported receiving insufficient support from clinics, particularly when it came to managing the transition back home. They would have liked, for example, more information about the personality changes (behavior and emotions) of their loved ones or support in assessing which tasks they could reasonably expect the persons living with stroke to handle. Without generalizing, these experiences do not seem to be unique to the Swiss context. For instance, qualitative studies from Malaysia, Denmark, and Australia have also described the need for information on comprehensive stroke care at home [34,35]. Apart from informal caregiver support groups, which did not appeal to the participating informal caregivers for various reasons (timing and setting), the informal caregivers were not aware of any other support services in the Swiss health care system. Because we exclusively captured the experiences and needs of 4 informal caregivers from the German-speaking part of Switzerland in this qualitative study, we plan a national survey as the necessary next step. It is a common practice [36-38] to first conduct a qualitative study, publish its findings, and then proceed with a quantitative study. In this survey, our aim is to expand upon the findings of this study and capture the needs of informal caregivers within the rehabilitation journey of their loved ones in more detail by involving a larger sample size and further regions of Switzerland.

### Accessibility to Quality Rehabilitation

Synchronous telerehabilitation following stroke experienced its initial upswing in Switzerland during the COVID-19 pandemic [39]. For the first time, in Switzerland, these services were covered by the basic health insurances for occupational therapists and physical therapists [40]. In our study, therapists reported minimal use, if any, of these services during the pandemic, while persons living with stroke did not use them at all. This contrasts with the results of a national survey, where >70% of occupational therapists reported providing telerehabilitation during the pandemic [39]. A crucial obstacle for the participating therapists was the existing uncertainty surrounding financial coverage. According to the current state of knowledge, since December 2023, it remains unclear whether the costs associated with synchronous telerehabilitation in the fields of physical therapy and OT will be reimbursed by the Swiss health insurances in the future. This highlights the need for a clear and enduring inclusion within the service catalog of Swiss health insurance providers, along with the effective communication of this inclusion. These findings show clear differences from other high-income countries in which technology-supported tools and telerehabilitation are already more established in the health care systems. In Canada and Australia, for example, intensive and promising efforts have already been made to implement telerehabilitation and the use of technology-based tools in outpatient rehabilitation [4,41].

### Adaptability to Patient Differences

Technology-based tools mentioned in the interviews and focus groups can be broadly categorized. These include tools specifically designed for therapy or training purposes, such as various apps and cognitive training programs for PCs or tablets. In addition, there are tools originally developed for everyday use, without initial therapeutic intentions, such as apps for public transportation, social media, and reminder functions on mobile phones.

Persons living with stroke who took part in our interviews and focus group experienced that the training tools developed for rehabilitation generally fulfill the necessary options for adaptability. However, they identified areas for improvement, especially regarding the shaping of technology, such as selecting appropriate levels and degrees of difficulty. Several persons living with stroke stated that the exercises were either too easy or too difficult, despite the tools automatically adjusting the difficulty level. However, the ideal difficulty level was often not achieved. Conversely, everyday technology devices such as smartphones, smartwatches, and tablets were commonly used. In addition, everyday apps such as reminder apps, communication apps, shopping apps, and public transport apps were frequently used. Most persons living with stroke stated that their use behavior often changed after the stroke. They used these tools more frequently. However, no cases were mentioned in which the apps or technologies used had been adapted. Nevertheless, for persons living with stroke, the use was usually possible with restrictions or only with the support of informal caregivers. A person living with stroke emphasized the importance of technologies with interactive features, such as technologies facilitating interaction with another person. This feature has the potential to enhance engagement by providing a role model or a trainer to demonstrate exercises.

### Accountability or Compliance With Rehabilitation

A key factor in successfully continuing the rehabilitation process following inpatient treatment with outpatient interventions is the willingness of persons living with stroke to take responsibility for their own rehabilitation process and to remain motivated to continue practicing independently [24].

The interviews and the focus group with persons living with stroke uncovered that technology-based tools can make a significant contribution to compliance in self-guided training at home. Particularly, the automatic visualization of progress and achievement of milestones were experienced as supportive. This enabled persons living with stroke to independently track their therapy progress and course.

In all cohorts, the relevance and importance of the self-responsibility of persons living with stroke were extensively discussed. There was a great consensus that independent and autonomous use of technology-based tools by persons living with stroke should be supported and forced. Past focus group studies conducted in Denmark have revealed similar attitudes [14]. They emphasize that technology-based tools can be viewed as an opportunity for persons to take responsibility for their own rehabilitation process. For the informal caregivers in our study, it was crucial, for example, that the persons living with stroke could complete their home-based training program independently of them. This provided some relief for informal caregivers, as they were not faced with an additional task for which they had to take responsibility. These experiences and perspectives may intersect with statements regarding the use of technology-based tools for persons living with stroke who are more severely affected. Specifically, some therapists argued that caregivers play a crucial role in the facilitation of home-based technology use for persons living with stroke with severe limitations. One potential avenue for consideration could be the recognition that clear communication between therapists and informal caregivers regarding responsibilities and the assumption of roles is important.

An additional perspective shared by all informal caregivers, which was not explicitly covered in the data analysis codes, was the significant role that technology-based tools play for them in their loved ones’ rehabilitation journey. As previously noted, informal caregivers often bear a high level of responsibility and burden. To cope with this, they use various strategies, including relaxation exercises facilitated by technology-based tools. These experiences underscore the potential of technology-based tools to support informal caregivers throughout the rehabilitation process. The diverse potential for the support of informal caregivers has also been highlighted in a rapid review [42], showcasing areas such as education, remote consultations, and reminders, which can be covered through the use of various technology-based devices.

### Engagement With Rehabilitation

It was particularly important for the participating occupational therapists that the use of technology-based tools added value to the patient’s everyday lives, which aligns with their professional profile. Ensuring this was described as a considerable hurdle. The therapists of both professions saw one way of overcoming this in anchoring technology-based interventions in tariff systems and establishing further training opportunities and recommendations on the part of professional associations. In Switzerland, occupational therapists and physical therapists are represented by professional associations. In addition to negotiating contracts for therapists with health insurance companies, the strategy of these associations is to further develop and train the profession and ensure quality assurance [43,44].

A key resource appears to be the exchange in professional networks and interest groups at the national level, which are also anchored in the professional associations.

Persons living with stroke use technology-based tools in Swiss outpatient stroke rehabilitation both to engage in their exercise programs or work on their recovery and to facilitate their daily activities. These uses often overlap. Some persons living with stroke reported using more technologies after stroke and even purchasing new devices (such as tablets or smartwatches). These people use these devices in their daily lives, particularly for compensation or training. The use of technology-based tools is strongly influenced by the limitations that persons living with stroke experience. This indicates a need for a high degree of adaptability of the corresponding tools. The group of persons living with stroke trusted and relied on the recommendations of their treating therapists when selecting suitable tools.

### Strengths and Limitations

This study possesses both strengths and limitations. We want to emphasize that the research team consisted of health care professionals, including 2 occupational therapists, 2 physical therapists, and 2 neurologists, all of whom are very familiar with the health care system in Switzerland. Moreover, 5 (83%) out of the 6 authors even possess several years of experience in clinical practice. This enabled them to compile and interpret the data results for the Swiss context appropriately.

The study presents some limitations, including a small sample size from 1 country and context. The findings only reflect the experiences of persons living with stroke, informal caregivers, and therapists in the German-speaking part of Switzerland. Further studies are needed to gather experiences from people of the Italian- and French-speaking parts of Switzerland. Furthermore, the focus of this study was on experiences using technology-based tools. We realized that in the interviews and focus groups, the informal caregivers reported on their experiences of how they perceive their role in the rehabilitation process and what they would have needed in detail. We tried to acknowledge these findings partly in our discussion. However, we could not delve into all aspects of these experiences, as these findings were beyond the codes and the scope of this study and were not explored and reflected in depth. Nevertheless, we consider it relevant to include the experiences of informal caregivers in the improvement of Swiss home-based stroke rehabilitation.

Furthermore, we were confronted with language-related issues. As described in the *Methods* section, the interviews and focus groups were conducted in Standard or Swiss German. The various dialects of the Swiss language have different grammar and sentence structures compared to Standard German. As we opted for a literal and simplified transcription and translation into Standard German and, subsequently, into English, some quotes may appear unfamiliar to native English speakers. To address this issue, we have added the original statements in Swiss German to the corresponding quotes in this paper.

### Practical Study Implications

For practicing occupational and physical therapists, the experiences and needs described by the 3 cohorts in this study can be used to reflect on their own practical experiences and perspectives. For example, a point of reflection could be the role and responsibility they attribute to informal caregivers and persons living with stroke regarding technology-based, home-based training. These considerations should also influence the selection and potential adaptation of technology-based tools in the home-based rehabilitation process after stroke. Furthermore, this study revealed that various conditions (funding, the selection of appropriate tools, guidelines, recommendations, etc) are not satisfactorily addressed for occupational and physical therapists within the Swiss health care system. This could motivate practicing therapists to actively participate, for example, in professional associations and in shaping further developments.

Persons living with stroke and informal caregivers who read this study may recognize themselves in the experiences of our participants and might feel a sense of belonging to these cohorts. Sharing these findings could also be enriching and valuable for others, such as informal caregiver support groups.

### Conclusions

The objective of this study was to describe the experiences of persons living with stroke, informal caregivers, and therapists regarding the use of technology-based technology in home-based rehabilitation within the Swiss context. Persons living with stroke, informal caregivers, and therapists had very different and unique experiences with the use of technology-based tools in this setting. It was shown that a broad spectrum of different tools is already available and is also being used. However, there remain uncertainties and ambiguities regarding financial reimbursement and education on the use of such tools in Switzerland. Furthermore, written recommendations for the use of technology-based tools in stroke rehabilitation are needed for the Swiss context. Clarification of these points could lead to greater confidence in the use of such tools, both on the part of therapists and on the part of persons living with stroke. With this research, we have illustrated the experiences and needs of our cohorts within the Swiss context. Therefore, conducting a national survey is the next step to depict the needs of informal caregivers in greater detail and breadth.

## Appendix

Multimedia Appendix 1 Quotes from study participants.

## Abbreviations

A3E: accessibility, adaptability, accountability, and engagement
ICT: information and communication technology
OT: occupational therapy
TA: template analysis
