# Supplementary material for: Technology Use for Home-Based Stroke Rehabilitation in Switzerland From the Perspectives of Persons Living With Stroke, Informal Caregivers, and Therapists: Qualitative Interview and Focus Group Study
Source: JMIR Rehabil Assist Technol. 2024 Jul 18;11:e59781. doi: 10.2196/59781 (PMC11294768; doi:10.2196/59781)
Supplement: Multimedia Appendix 1 [file rehab_v11i1e59781_app1.doc]

**Table 1.** Quotes from study participants

| **Category: Accessibility to Quality Rehabilitation** | | | |
| --- | --- | --- | --- |
| Subcategories | Quotes: Persons after stroke | Quotes: Caregivers | Quotes: Therapists |
| Facilitation of home-based tele-rehabilitation | S4: [has no experience with telerehabilitation] I saw something like this for the first time in a report on television.  S7: I think that [telerehabilitation] would surely make sense, but I don’t know if there’s an offer where I could do that. |  | T7: We can create a new exercise and simply film the person or us and then save it [in the telereha-tool]. And that has the advantage that is secure in terms of data protection law. […] I can specify that this patient can be seen in this exercise and then this exercise, only this patient can see it.  T6: During the pandemic, I also supported someone via Zoom through telerehabilitation. That was a young woman, who was mainly concerned with daily structure and discussing and organizing her everyday life.  T10: Another point was the tariff, so that it could be billed at all. That was also a bit of a struggle. […] We currently have a few private self-pay patients every week who are still using this service. |
| Education and training by the therapists | S7: The easiest way would be via the therapists. That they can say “This is available”, “this is the place where you can get it”. […] I think the therapists know what support is needed and what is available. | C3: I’m the kind of a person who thinks that there are experts for that. I think it’s important for us caregivers, we already take on so much, but that we don’t take on too much. Especially elderly caregivers. I already find our life so radically different; if we had to program the tool ourselves, it would be annoying. […] And I have realized, I can only speak for myself; it is much more effective if other people tell him something. […] That’s why I believe it’s more important for him to do it with therapists. | T2: If I may make a wish now, I would say, action recommendations. I think that would be good and helpful.  T10: I understand that it can be a bit complicated, that it takes time to learn. That there should be less resistance from the therapists themselves. But the resistance […] comes more because they have feeling that it’s voluntary work a bit. It’s more something extra to do rather than a helpful tool. |
| Education and training of patient | S6: I need low-threshold access to apps so that I don’t have to spend three days looking for something smart in the app store. I end up downloading something that I then buy, and I don’t really want that. I really want something of good quality that I don’t have to search for. […] It would certainly be great if the therapist had a list of apps, for example, that she or he hand out and say “Look, you can practice a bit of this here and a bit of that there”. | C3: I think mostly it would benefit me if there were a tool that would constantly remind him. Firstly, to provide a daily structure. But also, to remind him even just take a shower. It’s not just about appointments, but really about everything. | T2: Clients really like these exercises as a supplement. And if someone wants that, I think it's always good for basic motivation, even for everyday things, that someone who is fundamentally motivated also has the feeling that they are learning something with these apps, then I think that's really absolutely fine.  T5: For me, it's more a case of looking at what the patients are already using at home and trying to incorporate that. Some of them have smartwatches, for example. They're great for setting alarms or reminders and trying out new functions.  T10: This app for movement exercises. I've been using it for six years. For me personally, it is a very helpful tool for patient compliance. |
| Education and training of family members/caregivers |  | C1: My husband was in rehab, and he had a full schedule. He sent me that, then I saw what he was doing. People were taking care of him. And I was at home, had four children, one is going through a tough puberty. Then nothing happened. So, I didn’t receive any information about the meaning [of his health condition] and how he would behave when he returns.  C2: And to develop a tool that helps the caregivers to set boundaries somewhere and to know how far I can get involved. How far I can leave my husband a bit of responsibility. That he is also responsible for himself somewhere and does certain things. That’s a very, very fine line, but you have to learn it.  C3: And I know they [rehabilitation clinic] would have such caregiver course, but they didn’t offer it to me, even though the pandemic was still going on. | T6: I also involve the caregivers in the therapy as much possible, i.e., as much as necessary and as little as possible.  T1: I keep realizing that there’s a bit of potential for conflicts when relatives have to support the person after stroke in using their smartphone etc. […] I don’t like burdening relatives with such tasks either.  T9: When I think about it, I have the feeling that I would rather only give a home program to those patients who can really use it independently. I have the feeling that if a caregiver is needed to help to start the program. Then it’s probably just a bit too difficult overall. |
| Provision of technology-assisted after-hour therapy while inpatient for self-management of rehabilitation |  |  |  |
| Appropriate selection of technology | S6: I would be interested in downloading these things for training purposes. But I’m almost overwhelmed in the app store when it comes to downloading new things. It’s confusing, there’s a lot of ads and stuff. […] Because I can’t do that, reading and understanding a manual. Comprehension is no longer possible. […] Just something that is visually very appealing, something that is simple. I belong to this category; I don’t want to spend hours browsing around. I want to know quickly what it’s about. | C1: I’m of the same opinion as C2. I find online very pleasant because you don’t have to go away, you don’t need the time to travel. […] | T7: Usability must be good. Medical devices are often incredibly safe, but they are also incredibly complicated. And it has to be really, really […] simple and easy to break down. What makes an app ideal for me is that I can use the data that I collect there. I can integrate it into my practice information software, in other word into my practice system.  T6: It would certainly be user-friendly […] in our case it’s for people post-stroke. […] Each level should be captured from cognitive impairment, motor impairment, reduced attention, visual impairment etc.  T6: Last year we [T6 and T1] had a specialist meeting with the interest group technologies in occupational therapy, where I received lots of new ideas.  T10: Before we move forward with the development of technology, we need to make sure that the technology is accepted by our associations as part of the profession and that it can be billed appropriately. After all, there are great technologies, that we can theoretically already use in healthcare. But the barrier is the tariff, the political hurdles. |
| **Category: Adaptability to Patient Differences** | | | |
| Subcategories | Quotes: Persons after stroke | Quotes: Caregivers | Quotes: Therapists |
| Adaptability of technology (general) | S2: I had the feeling that it [ the rehabilitation process] takes time and that you have to accept it [own limitations].  S4: Attractive colors that I always like to see. […] Even better would be nice background music that you can perhaps select, that would be good. |  | T12: I also needed a lot of time at the beginning, […] until I became familiar with it […] It took a lot of clicks […] you sill have to individualize everything a bit. Yes, I was a bit afraid to really put it into practice. |
| Adaptability of technology to patient’s physical ability | S7: So for me it’s a challenge [using a smartwatch] because I can’t use my left hand very well. Then I always have to take my right hand and place it somewhere.  S7: At first I thought I would do the program from home. But at home it’s really difficult to start the PC until I have to log in and so on. Normally I have to press Ctrl+Alt+Del and that is not longer possible with one hand. |  | T6: But I was thinking that it would certainly need to be user-friendly […] You would need to look at what deficits might be present in our clientele was what is needed so […] each level can actually be captured from […] motor impairment. |
| Adaptability of technology to patient’s cognitive ability | S7: In the beginning [of the outpatient rehabilitation process] I needed support because I had forgotten many passwords or was not longer sure of them. And the I was locked. |  | T9: What I find more difficult with technology or computed-based training in general is that there are still a lot of people in neurorehabilitation who are too cognitively weak. Even though certain programs start at a basic level. Sometimes it’s still really difficult for them. […] [To the question if the person has tools to address patients that are severely cognitive impaired] Um, I don’t think it’s computer-based. Or I don’t know it. So I would work with other material. |
| Adaptability of technology to patient’s psychological ability | S1: […] after the stroke a little stress was enough […] I could lie down on the floor and sleep for half an hour, that’s how much capacity my head needs. |  |  |
| Facilitation of practice in severely impaired patients | S7: Or I can do a lot via language control, then it works “tip top”. […] This way I can write the text much faster than by hand. | C3: So, I clearly don’t know how elderly people [after a stroke] deal with technology. But a young person who is familiar with technology can of course do it [is expected]. Because in our case, the therapists have overestimated him. | T11: But basically, we try to involve the social environment as much as possible as a supporting factor. Otherwise, we don’t offer this now, but I think it’s definitely worth paying a visit to the home.  T11: The way to therapy is usually difficult, especially for severely affected stroke patients who cannot get in and out of the car quickly. It can therefore be a great relief if part of the therapy take place at home. |
| Promotion of progression to next level based on improvement | S2: I sometimes reached my limits there because you always had the feeling that you had to be fast. But that was never right for me.  S6: I would actually still like to do it, but it would have to be something simple. And then gradually increase it. |  | T11: For me [technology] would be rather cool for, let’s say, increasing the intensity of therapy. Because if someone is simply allowed to come to me once a week. Purely in terms of prescription, that’s relatively little. But if I could then use this application to make sure that the person does something twice or three times more a week without having to be with me.  T2: I really like the programs where the level adjusts a little. […] Then you reach the limit, and when you can’t do any more, it gets a bite easier again. […] Unfortunately, there are not that many such programs. |
| Provision of age-appropriate options |  |  | T3: The elderly patients said that [videoconferencing] was too complicated. We often just called them and asked how things are going and whether they performed their exercises […]  T1: I did a few videoconferencing sessions […], but they were all with younger patients after stroke.  T2: I think the cognitive training program […] Almost nobody can do that, maybe very young patients, where it’s about getting back into work. |
| Provision of interactive options | S4: Of course, it’s even more interesting when it’s interactive, when the trainer tells you afterwards that it was good […]. If someone could show me, that would be great. […] To practice, I naturally like to have a role model. […] When it comes to reaction, i.e. action and reaction between therapists and patient, it almost has to be online. |  | T11: If you design an app that it is a bit interactive, that would be nice. I think something like that would be important.  T7: We have an app with which we can send exercises. |
| **Category: Accountability or Compliance with Rehabilitation** | | | |
| Subcategories | Quotes: Persons after stroke | Quotes: Caregivers | Quotes: Therapists |
| Facilitation of self-management | S7: The exercises are on there [the app], and then I know whether I’ve done them or not. […] But I can also skip it, if I think I don’t feel like doing the exercise or something. | C4: What we did when she came home, we bought a Smartwatch with which she can make calls, even without a mobile phone. […] simply because if she falls somewhere or somehow, she can make a call.  C2: And something […] for the caregivers with very good instructions. For example, I always find breathing exercises very important for relaxation. […] That is very, very, very important at this time. | T9: Or perhaps edit a calendar together with the patients or set reminder functions and such things.  T10: My situation is like T12. We try to incorporate this [technology] into self-management, even if it is associated with limitations [of technology use]. I try to find a level at which the patient can still take responsibility for themselves.  T9: But when I think about it, I also have the feeling that I only give a home program to those patients who can really use it independently. |
| Monitoring of performance synchronously and asynchronously | S1: When I look at the timesheet, it was automatically activated from the start how long you were on duty.  S4: After half an hour, you will have an evaluation on the screen of what you did wrong, what was good and what progress you have made. That is certainly good and motivating.  S6: It is therefore inexplicable to me how it [the feedback] is created. I can’t understand the ratings. |  | T9: Yes, online. Accompany or be able to see when the patients have trained, for how long, etc. That you at least have the opportunity to talk about it. And I think that if they know that the therapist sees it, then it also has a different effect or perhaps motivates them differently.  Asynchronous then worked very well.  T10: And with virtual reality, the point is that the patient receives feedback and the therapist can exactly was being done. |
| Provision of feedback to patient synchronously and asynchronously | S7: If I haven’t gotten up once every hour from 8 a.m. to 8 p.m., it reminds me that I still must get up.  S4: Many people wait for feedback. Whether praise or criticism or correction, this is certainly very important. |  | T11: Because for gross motor skills […] it’s still difficult with a camera. Then you might have a little time delay or something. I found it difficult to give good feedback. |
| Enhancement of patient communication with providers | S5: [what the person misses when using technology in therapy] I miss senses of how the other person is feeling, perhaps also through a gesture, what the person wants to emphasize. All of this would be lost on the screen.  S6: I noticed it this morning, for example, with the cognitive training program. I had to tell the therapist during the exercise that I had done quite well. |  |  |
| **Category: Engagement with Rehabilitation** | | | |
| Subcategories | Quotes: Persons after stroke | Quotes: Caregivers | Quotes: Therapists |
| Facilitation of personally meaningful goal directed activities | S7: […] it always describes exactly what you want to achieve with this exercise and how exactly I must do it. And what I should pay attention to. It’s a bit like a movie, and that’s super. […] That’s also the good thing about technologies. I think YouTube is great for using things like that [ideas for everyday life strategies]. […] I also looked up how to zip up your jeans with one hand.  S6: I listen to a body scan as a prophylactic measure, if I know that something strenuous is coming up, I already do relaxation sessions. | C2: Mainly emails. Now I have a communication system with the health insurance company that uses encrypted emails. […] In the past, they always wanted me to send prescriptions and everything else by post, or I had to scan them. That was much more complicated. […] In the event of a stroke, the bureaucracy takes up almost as much time as the healing process. | T9: For me, it’s always a bit of the specific goal and that is simply always everyday-life oriented and individual at best. That’s why I’m also critical of technologies in the broadest sense, whether they can really do justice to the complexity of everyday life.  […] [Goal setting including technologies] usually rather limited, as they are always constructed settings or are modeled on a everyday life situation. […] I think that’s the main limitation, it’s always an imagined reality.  T4: What I always try to use are apps, calendar apps on the mobile phone, so that you can really concentrate on the activity. Or also the SBB app [public transportation]. I’ve practiced for hours with patients on reminder functions and apps, where you can make notes or something. The things that I also use myself. |
| Provision of positive feedback | S2: What annoyed me was that they [cognitive training program] said I was too slow. […]. I sometimes reached my limits because I always have to be fast. But that was never the right thing for me.  S1: I’m just incredibly happy when I make it under two minutes [and see this result]. | C2: I don't find online groups [for caregivers] that bad anymore because the information gets through, and support is also received.  C2: With apps like this [which provide feedback], there is a risk you will qickly think you have done something wrong. For him [her husband], it was a real panic, this so-called fear. | T11: So, if you can also display successes graphically, which is often possible with apps […]. The patients respond very well to it in the home application.  T12: It also has the function of […] motivating people when they can tick something off. Having completed something […] is also motivating for people.  T1: […] with reward systems and some “bling-bling”. “Hey now you have done this exercise”. Now treat yourself to a cup of tea […] How do you reward yourself and what’s good for you? |
| Provision of enjoyable and motivating interaction | S7: All of them [exercises] always come up. But I can skip it when I am thinking I don’t feel like doing an exercise.  S4: I could also imagine seeing myself as an avatar on the screen. Dressed like me, my face is there and would say “hello, I am your second self. Now I’ll show you what else you can do”. That would motivate me and also amuse me a little.  S4: If we can make the background attractive, we say that you can see moving pictures of Paris or something similar. |  | T11: I think it’s positive when it’s linked to a game. Such playful things are very well received. So people are usually very motivated to use them. |
| Facilitation of connection and feeling of community | S1: It was over one year before I met someone who had also experienced a severe stroke. He was also in rehab, and we now know each other well and go out for lunch together. I missed that.  S5: I wouldn’t do an exercise program in front of the computer and then move my arm or legs accordingly, that doesn’t suit me at all. […] I prefer dialog with people.  S7: I had never done these voice messages before. After the stroke, I realized that it was much easier for me than writing a text.  S1: We have five children. They’ve all clicked through [the cognitive training program on the computer] to see how fast they are [compared to me]. | C2: On the one hand, I was affected as a relative myself, and on the other, I learned a lot for my work and for what I am doing. There are so few books, so little input on how caregivers should really deal with these serious illnesses.  C1: [I would have liked] more information about what to expect [all the bad things that could happen]. If this is not the case, you can be relieved. […] But it would give me the feeling that don’t have to do everything on my own.  C2: Having all this as a relative is real work. But you can train and learn all of this. You can gain it all through new perspectives. […] It doesn’t have to be a group on social media, but a “video group” where you meet once every two or there weeks. And then you don’t just talk about how hard it is […] but: “I’ve experienced this and that, how do I deal with the situation when my husband has a hangover and doesn’t want to go to physiotherapy when he’s in pain?”  C1: I think there are support groups, but I don’t feel like it. I don’t want to be in a place where people call themselves victims and say, “We’re the poor ones”. I’d rather have a therapist helping me now, like I did, contributing ideas instead of just self-help.  C2: But now we know that all have a lot of commitments, things, or travel, and it has become more complicated. That’s why I’ve realized that it’s not so bad to just talk online. You can get into the topic very quickly.  C1: Our family doctor told me that everything that is still there six months after a stroke is preserved. That’s not true at all. And if you have a group […] you can share your experiences. | T1: Above all, communication, texting, to re-establish contacts [I have experienced this as relevant] |
